# Influences of Native American land use on the Colonial Euro-American settlement of the South Carolina Piedmont

**DOI:** 10.1371/journal.pone.0195036

**Published:** 2018-03-29

**Authors:** Michael R. Coughlan, Donald R. Nelson

**Affiliations:** Department of Anthropology, University of Georgia, Athens, GA, United States of America; Institut Català de Paleoecologia Humana i Evolució Social (IPHES), SPAIN

## Abstract

We test the hypothesis that prehistoric Native American land use influenced the Euro-American settlement process in a South Carolina Piedmont landscape. Long term ecological studies demonstrate that land use legacies influence processes and trajectories in complex, coupled social and ecological systems. Native American land use likely altered the ecological and evolutionary feedback and trajectories of many North American landscapes. Yet, considerable debate revolves around the scale and extent of land use legacies of prehistoric Native Americans. At the core of this debate is the question of whether or not European colonists settled a mostly “wild” landscape or an already “humanized” landscape. We use statistical event analysis to model the effects of prehistoric Native American settlement on the rate of Colonial land grants (1749–1775). Our results reveal how abandoned Native American settlements were among the first areas claimed and homesteaded by Euro-Americans. We suggest that prehistoric land use legacies served as key focal nodes in the Colonial era settlement process. As a consequence, localized prehistoric land use legacies likely helped structure the long term, landscape- to regional-level ecological inheritances that resulted from Euro-American settlement.

## Introduction

The process of Euro-American settlement of the South Carolina Piedmont, and elsewhere, established historically and spatially contingent land-use patterns that continue to influence the trajectories of social-ecological landscapes [1–4]. Ecological legacies from post-settlement land-use in Eastern North America are documented at the plot [1–4] and regional levels [5–7]. Settlers did not randomly scatter across the landscape, but rather, conditioned their choices to a set of criteria that reflected the demands of contemporary cultural, economic and agricultural systems. The land these settlers encountered was not pristine untouched wilderness, but a landscape that was shaped and modified by Native American populations for thousands of years [8]. In this paper, we argue that the settlement patterns and persistent legacies of the Euro-Americans were themselves conditioned by the land use legacies of the Native Americans that preceded them. To explain the significance of this patterning, we draw on the concept of ecological inheritance from niche construction theory [9].

Long term ecological studies demonstrate that land use legacies influence processes and trajectories in complex, coupled social and ecological systems [7, 10–13]. In Europe, where intensive agricultural land use has been practiced for millennia, the ubiquity of land use legacies is well established [14–17]. On a much smaller scale, ecologically significant land use legacies have been detected in South America [18–20] and in Mexico and Central America [21, 22]. Native American land use likely altered the ecological and evolutionary feedback and trajectories of many North American landscapes [8, 23–27]. Paleoecological and coincident archaeological data from some locations support this argument [28–31].

However, considerable debate revolves around the scale and extent of the land use legacies of prehistoric Native Americans. Some scholars argue that the ecological legacy effects of past Native American land use were significant and nearly ubiquitous [26, 32]. Others maintain that the effects were highly localized and therefore insignificant at broader scales [33–36]. At the core of this debate is the question of whether or not European colonists settled a mostly “wild” landscape or an already “humanized” landscape. Progress in eastern North America has focused on resolving issues of pattern and scale of prehistoric and historical Native American land use. This work suggests that localized patches of anthropogenic disturbance were embedded in a broader, heterogeneous landscape matrix [37, 38]. As a consequence, the vast majority of the prehistoric North American landscape was dominated by natural rather than anthropogenic forces. Even so, at any scale, empirical analyses of material legacies of past Native American land use in contemporary socioecological systems remains relatively limited [6, 39–41].

Here, we test the hypothesis that cumulative, environmental legacies of Native American land use influenced the location and timing of Euro-American settlement events for individual parcels of land in the landscape surrounding the confluences of the Broad, Enoree, and Tyger Rivers of South Carolina. We use an event-history analysis [42, 43] to statistically model spatial and temporal associations between archaeological evidence of prehistoric Native American land use and Colonial Euro-American settlement processes in the Southern Piedmont Physiographic Region of eastern North America. Historical narratives suggest that Native American agricultural legacies (successional old fields, floodplain canebrakes, semi-cultivated nut and fruit bearing trees) were important in the Euro-American settlement process [44, 45]. Given widespread acknowledgement of localized effects of prehistoric land use surrounding settlements [34, 38], we take archaeological evidence of Native American occupation as a proxy for the potential occurrence of prehistoric land use legacies. Such assumptions about land use (and their legacies) are also consistent with archaeological theory in central place foraging and settlement ecology which posits that land use intensity declines as the distance from residence and work sites increase [46–48]. Thus, legacies in soils and vegetation type and cover were most likely located in the immediate proximity of the residential and food processing sites evidenced by archaeological material.

Our results demonstrate the degree to which prehistoric archaeological sites served as key focal nodes in the Colonial era settlement process, thus tying emergent Euro-American land use and ownership patterns to land use legacies of prehistoric Native American farmers. We argue that our findings support the idea that for the South Carolina Piedmont, localized prehistoric land use in the immediate area surrounding prehistoric archaeological sites engendered long term ecological legacies that served as settlement opportunities for colonial Euro-Americans. These initial anchor points then structured subsequent settlement patterns from which contemporary regional-scale land use legacies derive. Thus, past cultural occupations can present subtle but persistent ecological opportunities and constraints in socioecological systems that influence the settlement ecology of successive occupations.

### Study area

**Fig 1:**
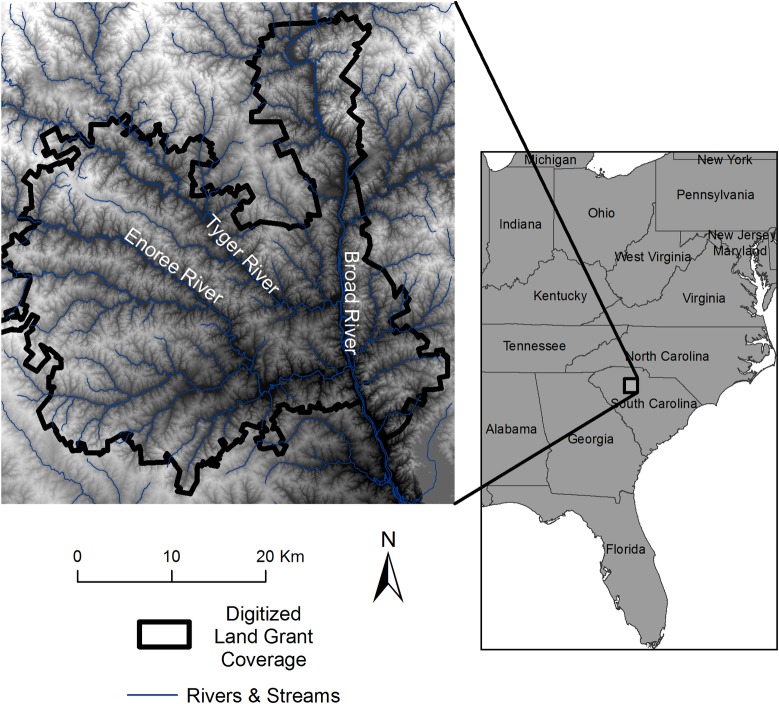
Location of project area.

Our project area is located at the on-going NSF funded research at the Calhoun Critical Zone Observatory (CZO), a collaborative, broadly interdisciplinary research group focused on the long term effects of historical land use on hydrology, geomorphology, biology, and biogeochemistry of the South Carolina Piedmont. Our project area, within the Enoree District of the Sumter National Forest, encompasses a 1256 km^2^ landscape incorporating portions of the Tyger, Enoree, and Broad River watersheds (Fig 1). The climate is classified as humid, subtropical with approximately 1250 mm of precipitation annually, hot summers and mild winters, with temperatures ranging approximately -5 to 40°C [10]. The vegetation cover is dominated by mesic second growth hardwoods in bottomlands, and secondary pine and mixed oak-pine forests on side slopes and uplands, nearly of which is reforestation of former agricultural lands. Soils are characterized by acidic, highly weathered Ultisols on the upland interfluves and more fertile soils occurring on alluvial bottomlands [10]. Uplands are highly eroded and the bottomlands are aggraded with a meter or more of sedimentation, as a result of linked processes triggered by 19^th^ and early 20^th^ century plantation agriculture.

#### Native American settlement and niche construction

Southeastern prehistoric archaeology is divided into four periods: Paleoindian ca. 14,000–10,000 BP (Before 1950), Archaic ca. 10,000–2,700 BP, Woodland ca. 2,700–1,000 BP, and Mississippian ca. 1,000–300 BP [49, 50]. The earliest evidence of human occupation and use of the project area begins with distinctive lithic material attributed to Paleoindian phase, at least 10,000 BP [50]. Early populations followed a land-extensive hunter-gatherer mode of subsistence. Any environmental legacies from this period, real or potential, are not well understood, though the depletion of Pleistocene mega-fauna through overhunting has been hypothesized [51–53].

The Archaic period is marked by a diversification in lithic technologies, including prevalence of grind stones, the presence of hearths and storage pits, signs of demographic growth, and, late in the period, the development of pyro-ceramic technologies, and the exploitation of cultigens [49]. By the late Archaic (ca. 5000–3400 BP), foraging groups began to cultivate domesticates in upland and riverine settings [54]. On the Southern Piedmont, agricultural land use probably began later, either during or after the middle Woodland period [50]. Groups were farming bottomland floodplains by the late Woodland and this activity intensified with the cultivation of *Zea mays* (maize) during the Mississippian period [50].

For many years the transition from hunter-gatherer to intensive agricultural production systems was understood as a culmination of reactive, one-way adaptations to changing resource and demographic conditions [55]. Niche construction theory, argues instead that intensification and the increasing diversity of subsistence resources resulted from proactive adaptation in places already productive [24, 56]. The theory posits that positive feedbacks between land use and environment can increase the reliability of targeted plant and animal resources. Through the management and manipulation of plants, animals, and land forms, this type of ecological engineering confers increasing returns over time as the outcomes of past activities accumulate to form new, and more desirable environmental conditions. Niche construction is the reciprocal reinforcement of ecological engineering through social and cultural changes that respond to the modified environment, including the emergence of livelihood-centered value systems, localized traditional ecological knowledge, and specialized cognitive understanding of resource distribution across time and space, all of which confer adaptive advantages [57, 58]. Thus human niche construction proposes that human evolution is subject to genetic, cultural, and ecological inheritance [9].

Smith [54, 59–61] laid out a detailed, empirical account for the role of niche construction in the co-evolution of Native American society and plant and landscape domestication in parts of the Eastern Woodlands of North American. His argument suggests that the development of agricultural societies relied on a long term dynamic process by which Native American land use “domesticated” the landscape thereby making it more conducive to agricultural land use and its complimentary settlement arrangements [24, 59]. The process of domestication involved the localized restructuring of plant communities in ways that removed undesirably vegetation, transplanted or spread the seeds of economically desirable plants, and conserved and cultivated nut- and fruit-bearing tree species [24]. As a result of these multi-generational efforts, Native American settlements became specific focal points for the concentration of economically valuable plant species, were strategically located in reference to managed game and fish habitats, and were structurally “engineered” to accommodate agricultural endeavors. The attractiveness of this ecological inheritance ensured that many settlements were occupied over multiple generations.

#### Native American abandonment and Euro-American settlement history

The exact period of Native American abandonment of domestic sites in the project area is unknown. Larger, intensively used bottomland settlements may have been abandoned as early as 500 BP. Three radiocarbon dates place occupation of major Mississippian sites in the project area between 1018–1634 calibrated CE (Oxcal 99.7% CI, dates from Green and Bates [62]). Desoto’s entrada (ca. 1539–1543 CE) bypassed the project area, looping around it on the southeast and north, so early historical evidence for its occupation remains elusive [63]. By the 17^th^ and early 18^th^ century, Cherokee most certainly used the area for hunting and gathering forays, but perennial occupation of the area remains unknown.

During the late 17^th^ and early 18^th^ century, Euro-American traders, slave raiders, hunters, and itinerant cattle herders penetrated the interior of the South Carolina “backcountry”. The vicissitudes of the colonial economy and intermittent conflicts with the Cherokee and other Native American groups rendered their presence ephemeral. Although African-Americans certainly played an important role in South Carolina during both the Colonial and Ante-Bellum periods, for a variety of reasons, their presence in the backcountry was insignificant until the 1790s [64].

Early settlement in South Carolina focused on the coast and settlement in the Piedmont backcountry did not begin in earnest until the 1730s following a survey and map by George Hunter [65]. Soon afterward, townships were laid out that served as support bases for the settlement of the backcountry [65, 66]. Settlement was slowed due to numerous conflicts with Native Americans. Eventually, a 1747 treaty with the Cherokee and a garrisoned fort at the confluence of the Broad and Congaree Rivers opened up the Broad, Enoree, and Tyger River watersheds for settlement [66, 67].

Formalized legal agrarian settlement in British controlled portions of North America involved the institution of the “headright grant”, a process that transferred title of a specific parcel of land (the “land grant”) from the state to the individual [68]. During the colonial period in South Carolina (1663–1775), headrights were granted by the King of Great Britain. The first land grants for the watersheds included in this analysis were recorded in 1749. Grants ceased during the American War of Independence (1775–1783), but in 1784 the new State of South Carolina began to issue grants under the authority of the Governor. To receive a land grant, the settler obtained a warrant from the governor for a certain number of acres at a price of one half penny sterling silver per acre. Following this, the surveyor general measured and platted a tract of unclaimed land using the metes and bounds system [68, 69]. In contrast to the grid systems employed in the 19^th^ century settlement of the western United States, the earlier metes and bounds surveys fixed boundaries of tracts of various sizes and shapes based on compass bearings and the “Gunter chain” (1 chain = 20.1 meters) measure of distance. Early tracts, surrounded by vacant land, were located based on their relative proximity to named rivers and creeks. Later, where grants had already been made, their shape and location was indicated based on the cardinal direction by which they were “bounded” by neighboring lands (e.g. … bounded on the Northeast by Smith, on the Southwest by Johnson, etc.).

The land grant record shows an initial trickle of settlers from South Carolina and abroad. Settlement began to intensify with an influx of Scotch-Irish refugees from Pennsylvania and Virginia following the 1755 defeat of General Braddock at the hands of the French and Native forces [70]. Conflicts with Native Americans continued into the 1760s but by the mid-1770s the settlement “frontier” had moved west [71, 72]. However, land grants continued to be issued in the study area through 1851, filling in unclaimed land and reallocating tracts lacking clean chains of title.

## Material and methods

To test whether prehistoric Native American land use affected spatiotemporal patterns of Euro-American settlement we choose a Cox proportional hazard model (Cox model) [73]. Cox models have been successfully employed in similar types of event analyses [74, 75] and perform well with continuous and categorical (binary “dummy”) variables [43]. Event analysis examines the cumulative timing of a change in qualitative state. We represent settlement "events" as the date of first Euro-American claim to a specific parcel of land, evidenced by maps and legal descriptions for surveyed land grants. The analysis accounts for time such that rates of claims to land (by year), can be assessed with reference to fixed-time covariates representing the physical characteristics of the parcel of land being claimed.

Analysis relied on three main sources of data: (1) historical land grant survey maps with associated grant dates, (2) archaeological survey and site locations with relative dates of occupation; and (3) a 10m^2^ digital elevation model (DEM) from the USGS. We used a GIS geodatabase to organize and sample these data for our statistical analysis of the timing of settlement events. Statistical analysis was conducted using Stata 11.2.

### Land grant and settlement geodatabase

The GIS land grant geodatabase was primarily derived the United States Forest Service (USFS) land purchase records for the Sumter National Forest created in the 1930s. The National Forest land purchase was well documented and consists of survey maps and legal descriptions stretching back to the original land grants. We cross-checked the USFS data with a local heritage atlas of land grant plats and other historical points of interest created by the Union County Historical Society in the 1970s [76]. For each tract of land purchased during the 1930s and 1940s, the USFS made a thorough effort to trace property ownership and legal bounding from its initial granting to the time of public purchase. The resulting dossiers for each tract of land included maps with surveyed property boundaries overlaid on the original survey plats associated with the land grants. The maps were produced for the Forest Service by professional surveyors and were accompanied by documentation for the chain of ownership beginning with the land grant text itself. Where USFS records did not provide data (approximately 13% of the study area), we used the Union County Historical Society Atlas.

**Fig 2:**
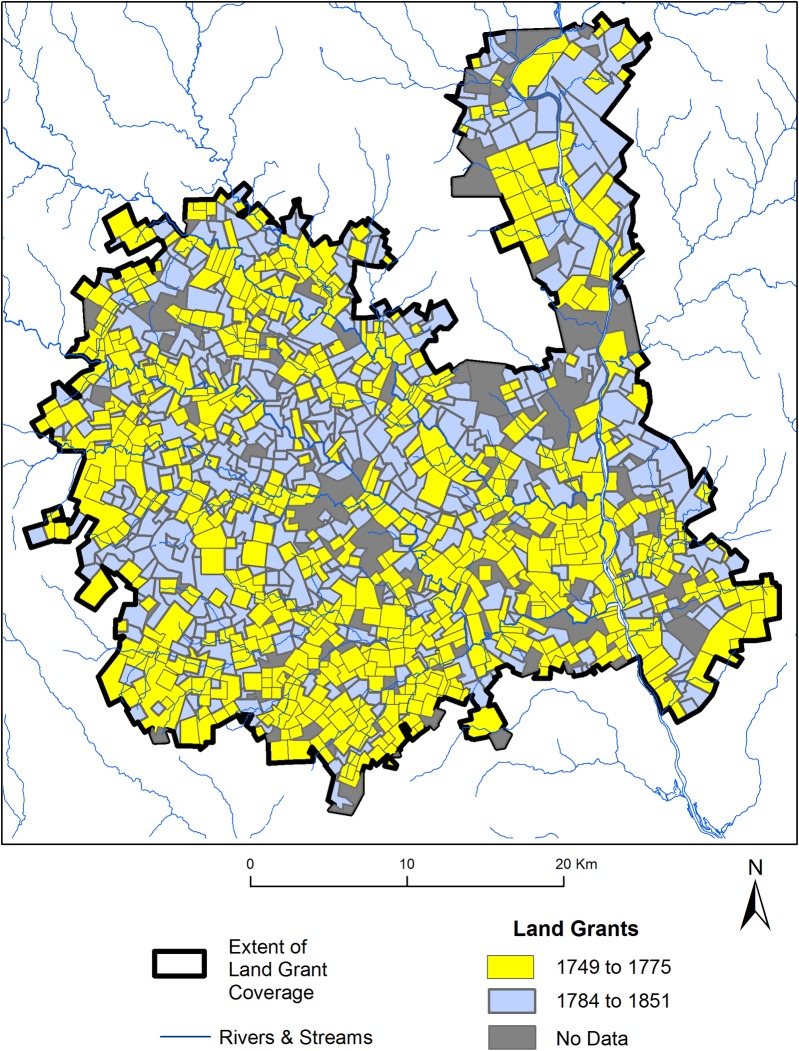
Land grant dataset and distribution within the project area.

**Fig 3:**
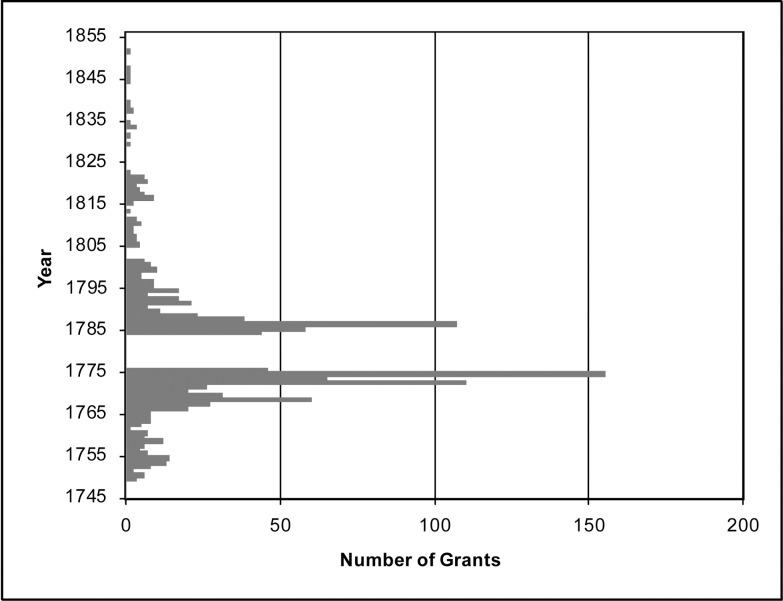
Number of land grants by year.

Where grants overlapped (e.g. a grant in 1765 incorporated into a 1784 grant), we digitized first record. In other words, where the area of a land grant was previously deeded, the surface area of the grant is only represented in the map where portion of the tract was not previously claimed. Our completed dataset includes 1,160 land grants each with year of survey, acreage granted, and the name of the grantee (Fig 2). Grants were distributed between 1749 through 1851. We divide this range into two settlement periods, which demonstrate distinct characteristic. The first ranges from 1749 to the onset of the war in 1775, and the second begins with the end of the war in 1784 through 1851, when the last grant was assigned (Fig 3).

**Fig 4:**
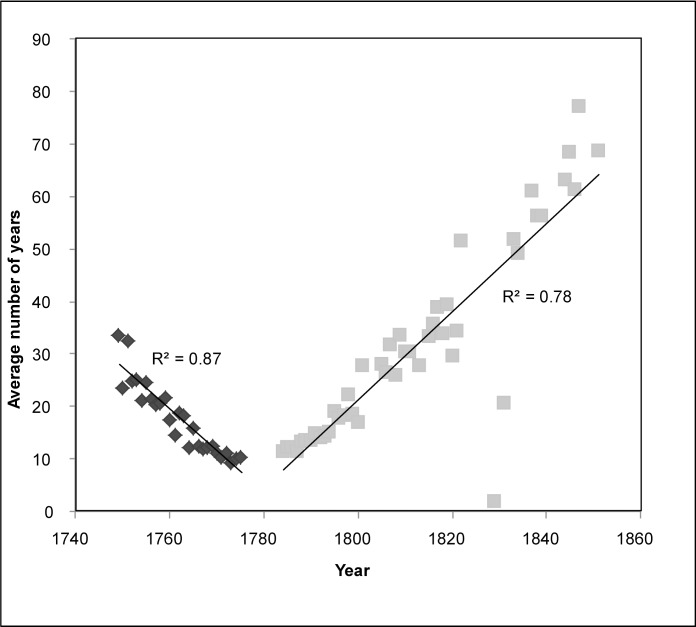
Average number of years between neighboring land grants by year of land grant.

We anticipated that the location of each land grant could influence the timing of successive neighboring settlement. To explore the spatial pattern and timing of settlement parcels with reference to their neighbors, we calculated the average number of years between a grant and its neighboring grants. When plotted by year of grant, the average number of years between a grant and its neighbors decreases for the initial settlement period. When granting resumed in 1784, the trend shows the inverse: increasing difference in years between a grant and its neighbors. These trends show statistically significant and correlatively robust linear associations (Fig 4). This patterning may indicate that the first division of the land sensu [77] was characterized by dispersed settlement. If settlers showed a preference for dispersion over clustering, we would expect to see higher average difference in years for earlier settlement and lower difference in years for later settlement. To account for these preferences in our event history model, we added a parameter of the average difference in years between a grant and its neighbors.

#### Settlement forts

**Fig 5:**
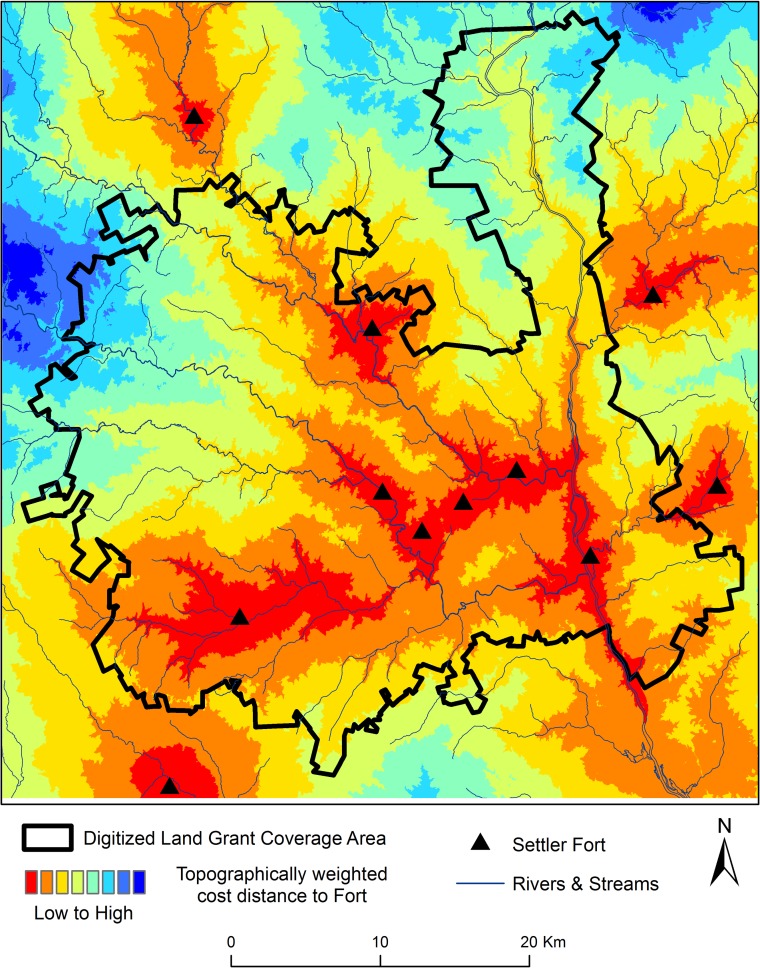
Location of Anglo-Cherokee War era settler forts and topographically weighted cost distance to nearest fort.

Fortifications against attacks by French and Native Americans were crucial all along the 18^th^ century Anglo -dominated settlement frontier [72]. In the South Carolina “backcountry”, inland of the Piedmont-Coastal Plain fall-line, domestic defense took the shape of settler “forts”, private homesteads fortified with stockades or palisades, *abatis* (a row of trees with sharpened tops), and Garrison blockhouses with gun portals and cantilevered overhangs [65, 78]. Indeed, settlers along the Enoree, Tyger, and Broad Rivers relied heavily on settler forts during the years 1760–61, the height of the Anglo-Cherokee War (part of the global conflict known as the Seven Years’ War) [66]. Further, in the years following these conflicts, lawlessness and chaos persisted in the South Carolina backcountry [67]. This history suggests the real possibility that concerns for safety and security influenced the settlement pattern and process. Consequently, we included travel cost-distance to settler forts as a covariate in our statistical model of settlement events. We used a combination of historical narratives, land grants, and archaeological site records to locate 11 forts dating from at least 1760–61 (the Anglo-Cherokee War period) (Table 1.). We then conducted a cost-distance analysis using the slope-weighted least-cost pathway between a land grant center point and the nearest settler fort (Fig 5).

**Table 1:** Source material for georeferencing the Ango-Cherokee war period settler forts.

Fort Name	Historical Narrative	Land Grant Name	South Carolina Archsite	Total Number of Source Types
Otterson’s Fort	Yes	No	Yes	2
Musgrove’s Fort	Yes	Yes	No	2
Pennington’s Fort (Isaac)	Yes	No	Yes	2
Pennington’s Fort (Abraham)	Yes	Yes	No	2
Gordon’s Fort	Yes	Yes	Yes	3
Aubrey’s Fort	Yes	Yes	No	2
Lyle’s Fort	Yes	Yes	No	2
Fletchall’s Fort	Yes	No	No	1
Waggener’s Fort	Yes	No	No	1
Wofford’s Fort	Yes	No	No	1
Brooks/Rhall’s Fort	Yes	No	No	1

#### Physiographic covariates

Physiographic factors such as topography and distribution of landforms exert important controls on land use and land use change [79–81]. We tested a total of 12 different physiographic factors. Quantitative and qualitative physiographic variables were derived from a USGS 10m digital elevation model (DEM) using the standard ArcGIS Spatial Analyst toolbox, the Geomorphology and Gradient Metrics toolbox, and Topography Tools (Table 2). Although the elevation range in our project area is relatively narrow (28–242 masl) we nevertheless included it since elevations are lowest toward the southeastern portion of the landscape and highest in the northwestern portion. Thus, we hypothesized that the effect of elevation on settlement would be indicative of the rate of settlement dispersal throughout the area. We included percent slope since we hypothesized that the flattest areas would be the most desirable and therefore settled first. We were interested in slope-aspect since settlers could choose south and east-facing slopes for agricultural purposes or north and west facing slopes for shelter from extreme weather such as hurricanes.

**Table 2:** Environmental and topographic parameters.

Attribute		Variable Type	Source/Citation
Elevation	Meters	Continuous	USGS DEM
Slope Degree	Inclination degrees	Continuous	ArcGIS Spatial Analyst Tools
Aspect (reclassified into 4 cardinal directions: North, East, South, West)	Degrees: N = 315–44, E = 45–134, S = 135–224,W = 225–314	Binary, presence/absence for each direction	ArcGIS Spatial Analyst Tools
Solar Radiation Index	Low = shaded, High = Exposed to Sun	Continuous	ArcGIS Spatial Analyst Tools
Topographic Wetness Index (TWI)	Low = dry, High = wet	Continuous	ArcGIS Topography Tools [83]
Site Exposure Index	Low = Sheltered, High = Exposed	Continuous	ArcGIS Geomorphology & Gradient metrics
Distance to River	Meters	Continuous	Calculated distance to nearest 100m spaced node on highest order rivers and streams
Topographic Position Index (TPI)	100m neighborhood Reclassified into 3 classes representing ridge top, narrow valley, and wide flood plain	Categorical	ArcGIS Topography Tools: Slope position index [83]

We also tested topographic and distance indices. The topographically derived solar radiation index and topographic wetness index capture factors important for agriculture: potential availability of sunlight and soil moisture. For example, Jones and Ellis [82] found that in comparison to other locations, long term prehistoric settlements in North Carolina had higher solar radiation values and well-drained soils. The site exposure index accounts for the possible preference for shelter from weather or visibility relating to site defensibility. The “distance to river” variable, calculated as the distance to the highest order drainages, permits analysis of the relative importance of the rivers to settlement. In addition to provisioning water, energy, and biological resources, river corridors connected the settlers to the coastal plain via water transport. Further, river crossings were strategic nodes along the important trade routes traversing the regions. Lastly, we included three binary variables representing the dominant landforms on the landscape: wide floodplain bottomlands, narrow valleys, and upland ridgetops, to assess whether settlers had preferences for particular landforms. The topographic position index (TPI) discriminates landforms based on the topography of a sample area’s neighborhood. We reclassified and simplified the TPI into three binary variables representing presence and absence of each landform.

#### Archaeological sites and pedestrian survey data

Archaeological survey, site locations (ArcGIS shapefile polygons) and reports for the area were downloaded from the South Carolina Site Files website (http://www.scarchsite.org). We relied on field assessments for determination of archaeological phase. For the analysis, we sorted the sites into four relatively dated, mutually exclusive categories (Table 3): (1) Long term prehistoric agricultural phase sites (ArchLongTerm) represented by sites with a Mississippian phase and a Woodland Component; (2) Late prehistoric agricultural sites (ArchMiss) represented by sites with a Mississippian phase component only; (3) Non-Mississippian prehistoric site (ArchNoMiss) represented by relative dated prehistoric sites with no Mississippian component; (4) Prehistoric sites of undetermined phase(s) (ArchUnknown) represented by lithic scatters with no temporally diagnostic artifacts. According to surface survey and systematic shovel testing, artifact densities at most sites were relatively low and relative dating was often based on 1 or 2 ceramics or projectile point styles. Consequently, our typology is based on presence of diagnostic artifacts rather than their density.

**Table 3:** Summary of sample units in comparison to archaeological sites in sample.

Parameter	Description	N 1-ha (sample units)	Archaeological Sites	Site Density
ArchLongterm	Mississippian with Woodland component	67	20	0.001
ArchMiss	Mississippian component only	112	36	0.002
ArchnonMiss	Diagnostic non-Mississippian prehistoric site	1037	328	0.022
ArchUnkown	Non Diagnostic prehistoric site	3080	1400	0.092
NoArch	No Archaeology Found	10,928	0	-
**Total**		**15,222**	**1,784**	**-**

#### Sampling

Individual archaeological sites in Sumter National Forest average about 4,800 m^2^ (standard deviation of 11,700 m^2^). Land grants, on the other hand, average 970,000 m^2^ (standard deviation of 866,000 km^2^). To reconcile differences in scalar resolution of our data and to adjust for any spatial biases resulting from spatial pattern and scalar-mismatch, we created an arbitrary grid of 100 x 100 m (1 ha) sample units represented in the geodatabase by a topology of polygons.

**Fig 6:**
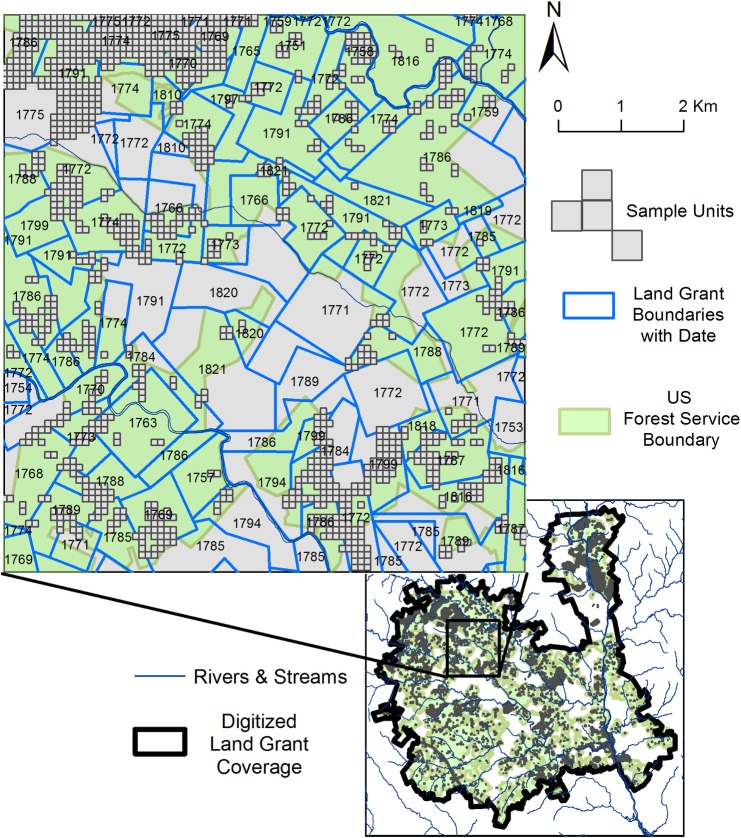
Sample strategy. 100 m^2^ sample units with land grant boundaries and modern US Forest Service boundary.

We limited our sample to units with a known land grant date that intersected areas systematically surveyed for archaeological artifacts and features (survey areas and recorded archaeological sites) (Fig 6). Other spatial studies of settlement have employed random sampling to control for the potential autocorrelation of archaeological sites with environmental attributes [82, 84]. However, since the presence or absence of archaeological evidence remains uncertain for areas that have not been systematically surveyed, we were concerned that random sampling could introduce errors. To eliminate this potentially confounding source of uncertainty, we sampled all systematically surveyed areas within the boundaries of our land grant coverage. Additionally, a large percentage of our sample area has undergone systematic subsurface archaeological shovel testing, thus improving our confidence in the accuracy of presence of archaeological material, its spatial boundaries, and the cultural period determination based on established chronological typologies of artifacts [50]. We extracted the physiographic and settler fort cost-distance covariates by calculating their mean value for each 100m^2^ sample unit. We created binary dummy variables for each of the four pre-historic land use categories to represent their presence (1) or absence (0) within the unit. Based on this sampling criteria, about 28% of our sample units show presence of archaeological material while the remainder is surveyed area lacking observed archaeological evidence.

### Cox proportional hazards model

In the Cox model, each sample unit is a uniquely identified case with records for each year of its “survival” up through its “failure” event [42, 43, 73]. Thus, time is represented on an annual resolution with the year of the first grant in our study area (1749) is represent as time 1 (t_1_) while the year of the last grant (1775) is represented as time 27 (t_27_). We use the year of the land grant (t_i_) as the time of Euro-settlement event. As our 100 m^2^ sample units are claimed by the settlement event, they “fail” and exit the analysis.

The Cox proportional hazards model is expressed as:

$$Log\;h_{i}\;(t)=a\;(t)+\beta_{1}x_{i1}+\cdots +\beta_{k}x_{ik}$$

Where a (t) is the survival time, and β_1_ through β_k_ are the coefficients for *x*_1_ through *x*_k_ covariates.

In our results, we report the proportional effects of each covariate on the rate of event occurrence as a hazard ratio. The Hazard ratio can be interpreted as the percent change in event rate given a one unit change in the covariate; results of 1 indicate no effect, > 1 indicate a positive effect and < 1 indicate a negative effect on the event rate. Thus, a hazard ratio of 1.25 indicates a 25% increase in the rate of event occurrence given an increase in the covariate value and a hazard ratio of 0.85 indicates a 15% (1–0.85) decrease in the rate of event occurrence given an increase in the covariate value. Statistical significance was determined based on a p value of <0.05 and the 95% confidence interval where the interval does not contain a value of 1. If the CI includes 1 (e.g. 0.95–1.25), this indicates the possibilities of negative, positive, or no effects.

Although we were only interested in the initial settlement period or first division of the land (1749 to 1775), we nevertheless modeled grants the period from 1784 to 1851 so that we could compare any differences in settlement preferences. To do this, we grouped the samples into two periods: period 1 samples with grants issued during the colonial era spanning from 1749 to 1775 and period 2 samples with grants issued from 1784 to 1851. We modeled each period separately and report the respective results below. We ran multiple iterations of the model and variously culled predictor covariates that were insignificant. The data used in the model is available in the supporting information files (S1 Table).

## Results

### Period 1: 1749–1775

The location of the prehistoric agricultural period sites and wide flood plains showed the strongest positive effect on the location and timing of Period 1 (Colonial era) land grants (Table 4.). Sample units with long term or recurrent prehistoric agricultural period occupations showed the strongest statistically significant effect with a 243% increase in rate of Euro-American settlement in comparison to units without such sites. Settlement showed similar increased rates for wide flood plains at 214% and areas within 100 m of the widest floodplains (29%). Units with Mississippian sites lacking a recorded Woodland component showed a 69% increased settlement rate. Finally, non-Mississippian sites showed a 50% increase and non-diagnostic lithic scatters showed a 28% increase in settlement rate. A one unit increase in TWI provided a 11% increase in settlement rate, showing the influence of wetter, bottomland soils. The average difference in years between neighboring grants was also slightly positive with a 3% increased rate.

**Table 4:** Cox proportional hazards model results.

**Period 1: 1749–1775**				
**Covariates**	**Haz Ratio**	**P>z**	**95% Conf. Interval**	
ArchLongTerm (Mississippian with Woodland)	2.426	0.000	1.958	3.222
Wide Floodplain	2.136	0.000	1.896	2.951
ArchMiss (Mississippian without Woodland)	1.689	0.000	1.416	2.071
ArchnonMiss (Not Mississippian, relative dated)	1.501	0.000	1.382	1.619
Within 100 m of wide floodplain	1.290	0.000	1.159	1.437
ArchUnknown (Prehistoric, no diagnostic)	1.284	0.000	1.212	1.350
TWI	1.087	0.001	1.041	1.182
Average Difference in Years	1.027	0.000	1.026	1.030
Site exposure	0.905	0.000	0.903	0.970
Elevation	0.992	0.000	0.990	0.993
River Distance	1.000	0.009	1.000	1.000
Solar radiation	1.000	0.000	1.000	1.000
Fort cost distance	0.000	0.000	0.000	0.000
**Period 2: 1784–1851**				
**Covariates**	**Haz Ratio**	**P>z**	**95% Conf. Interval**	
Within 100m of wide floodplain	1.465	0.001	1.160	1.849
ArchnonMiss (Not Mississippian, relative dated)	1.146	0.023	1.019	1.290
ArchUnknown (Prehistoric, no diagnostic)	1.098	0.004	1.029	1.171
Average Difference in Years	0.941	0.000	0.938	0.944
Site exposure	0.950	0.000	0.931	0.969
Elevation	0.991	0.000	0.989	0.994
River Distance	1.000	0.004	1.000	1.000
Fort cost distance	1.000	0.000	1.000	1.000
Solar radiation	1.000	0.000	1.000	1.000

Site exposure was a negative influence with a 10% decrease in settlement rate. Elevation showed a slight negative effect with 0.8% decreased settlement rate. The influence of the cost-distance to settler forts was also negative, indicating a preference to locate closer to these forts, however, the hazard ratio value was negligible at less than 0.001. Similarly, the hazard ratios for distance to rivers and for solar radiation were also negligible.

### Period 2: 1784–1851

The greatest significant and positive influence on land grant location and timing for period 2 was the 100 m proximity to wide floodplains (hazard ratio of 1.465). Wide floodplains themselves were dropped out of the model as they were not significant. Following this, the non-Mississippian sites and non-diagnostic lithic scatters showed relatively weak positive influences with a 15% and 10% increased rate respectively. Land grants were negatively influenced by site exposure (5% decrease in settlement rate). In contrast to period 1, the average number of years between a grant and its neighbors had a negative effect (6% decrease in rate for every 1 year increase). Thus, period 2 grants were more clustered in space and time than the proceeding period. Elevation also continued to exert a weak, but negative influence on settlement rate (1% decrease). Distance to rivers and cost-distance to settler forts were both positive, but negligible in hazard ratio value.

## Discussion

### The Settlement process

Our analysis demonstrates that the earliest Euro-American settlers had a clear preference for sites with a long Native American occupation history located on wide floodplains. Research suggests that late prehistoric agricultural people in Eastern North America were attracted to ecologically productive areas [82, 84]. A parsimonious explanation for the observed relationships between prehistoric Native American and Euro-American settlement is that for each phase of settlement, people selected the most productive areas from the ecological template. However, as our analysis shows, Euro-American settlers were not uniformly attracted to Mississippian period sites, they were more attracted to the sites with the longest and most intensive histories of use. Critically, these qualities are more significant than the environmental covariates hypothesized as important. As other studies have highlighted, Mississippian communities did not select their settlement locations based on environmental factors alone [84]. Indeed, whereas settlers showed a preference for proximity to wide flood plains in both periods examined, by the second period, prehistoric Native American agriculture sites were unimportant or were already claimed.

Settlers clearly valued agriculturally productive fields and grazing commons over proximity to neighbors or defensive fortifications. Despite the dangers apparent on the South Carolina Piedmont frontier, settlers followed a dispersed pattern. If the Piedmont had been predominantly “wild”, settlers would have been smart to aggregate around particular toeholds as they domesticated the landscape. Instead, they were able to spread out, giving priority to sites previously occupied by Native Americans.

As settlement density increased, settlement inevitably clustered and differences in years between neighboring land grants declined. These results thus confirm earlier observations that the frontier had moved farther west by 1775, likely leaving only land of the lowest agricultural value as common property. After 1784, land grants were constrained by the previous wave of settlement, with colonists selecting the lower value lands passed over in the first division. As has been shown for other areas in Eastern North America, later grants were “infilling” the landscape, as land owners began claiming the lower value, odd shaped parcels left behind by their predecessors [4]. Indeed, the land grant period 2 (post 1784) covariates show considerably diminished strength and significance in comparison to those of period 1. Rather than a second wave of settlement, the post-Revolutionary War second division grants probably represented efforts by existing residents to claim commons adjacent to previously improved lands.

Numerous legacies of Native American land use were observed historically in the Southeastern Piedmont, some of which are still in evidence today. These include extensive trail networks, previously cleared agricultural fields (“Indian old fields”) [66], fruit and nut tree occurrence [40, 41, 85], and fish weirs [71]. We hypothesized that these behaviors resulted in an unevenly distributed set of ecological conditions that were later conducive to the Euro-American modes of production at the time. If our hypothesis is true, settler preferences for these sites resulted in spatial patterns partly pre-determined by past Native American land use decisions. We therefore argue that Native American land use legacies laid the foundation for a landscape architecture that funneled settlers to key locations that became the focal nodes for the dispersed settlement strategy followed during the Colonial era (period 1). These initial settlement patterns then operated as historically contingent spatial constraints on the land grants that followed the Revolutionary War (period 2). Based on historical observations discussed below, we speculate that expediency and enhanced productivity were the two most important reasons Euro-Americans gravitated to areas characterized by Native American legacies.

### Expediency and “Indian old fields”

If optimal use of land involves maximizing productivity while balancing the ability to defend yields, land use decisions that eschew optimality and security in the face of labor costs or time constraints can be considered “expedient”. Agricultural settlement often requires expediency over optimality since time and labor are both limited during the construction the agricultural niche. Yet, as noted above, Euro-American settlement era landscapes of Eastern North American were hardly pristine. These landscapes had seen a form of agricultural niche construction before: historical maps and place names suggest that “Indian old fields” (abandoned cultigen fields) were fairly common landscape features [86–88]. Indeed, old fields are often mentioned in historical narratives as attracting early Euro-American settlement since these were easiest to clear and plant [44, 66, 89, 90].

Even though our study area may have been agriculturally abandoned by Native Americans perhaps as much as a century prior to the arrival of Euro-American settlers [50], the Cherokee, Catawbas, Euro-American hunters, trappers, and cattle herders continued to maintain an anthropogenic influence on the landscape [66, 67, 71, 91]. Mississippian trails and old fields likely provided a relatively low maintenance landscape infrastructure for the itinerant hunters and trappers. The seventeenth and eighteenth century deerskin trade between Native- and Euro-Americans had wide ranging social and environmental significance [44]. By 1750, the lower Cherokee villages were at least 100 km to the west of the project area, however Cherokee hunters exploited considerable geographical area beyond the immediate confines of their village territories [92]. They transported the deerskins to Charleston, likely traversing the Sumter National Forest area. Many Native American groups have been documented using fire to drive game [25, 93] and paleoecological evidence for increased fire activity in the early to mid-1700s has been attributed to the Southeastern deerskin trade [94]. Intentional and casual use of fire for hunting and to clear trails and campsites could have easily maintained patches and corridors of open landscape [95]. Historical geographer Prunty [96] (p165) hypothesized that “maintenance of open woodlands, via recurrent fires, and of old clearings ‘inherited’ from the Indians, was vital,” to the ephemeral cattle herding economy that preceded the agrarian settlement frontier. This fire maintenance hypothesis is also consistent with historical period descriptions of the settlement period landscape which characterized the Piedmont as extensive woodland savannah and prairie ridges divided by vast brakes of cane [91].

### Productivity and the canebrake

Cane (*Arundinaria gigantea* (Walter) Muhl.), a species of native bamboo, typically grows in extremely dense, monodominant stands called canebrakes that were a common feature of the settlement era bottomlands [45, 90, 91]. Canebrakes tend to grow on alluvial terraces as they tolerate moderate flooding. They are an early successional species, maintained by regular (~10 year) cycles of fire, and die out under conditions of fire exclusion or overgrazing [45, 97]. Historical narratives frequently noted the importance of canebrakes to the settlement process [66, 71, 91]. The livestock-dominant subsistence strategies of Euro-American settlers benefitted from A. *giganteana* because it is a highly productive forage crop and retains its nutritional value during the winter season when native grasses are dormant [90]. Settlers apparently judged the fertility of a site by the height of its cane (up to 12-14m tall) [91]. Indeed, A. *giganteana* does attain its greatest height in the most fertile soils [97].

*A*. *giganteana* was also extremely important to Native Americans, frequently appearing in regional ethnobotanical records of the Late Woodland period [98]. Canebrakes were likely propagated and managed as an integral component of late prehistoric Native American agro-ecology [45, 99]. The economic utility of A. *giganteana* makes it a good candidate for fallowing nutrient exhausted fields and for occupying less productive field edges and corners. Not only did A. *giganteana* provide material for basket-making and the construction of shelters, it likely played an important soil-amending role in the shifting agricultural systems employed by late Woodland and Mississppian peoples [90]. Periodic flooding in bottomlands deposits soil sediment that helped maintain the fertility in the prehistoric shifting agricultural system [87, 97]. Since A. *giganteana* grows in dense thickets, these can slow floodwaters, thus capturing more sediment than a typical stand of bottomland hardwood trees. When an old field was again needed for agriculture the cane could be harvested for use. If cane stems and slash were then burned, the residual charcoal and potash would provide additional nutrients to the soils. Over the centuries, cycles of maize cultivation and cane-fallowing might have improved rather than degraded the bottomland soils. Indeed, Ethridge [90] posits that invasive A. *giganteana* may have been a more important factor than nutrient exhaustion in prompting Native American riverine farmers to abandon and rotate fields. Occasionally clearing canebreaks for new fields would have been less labor intensive than hoeing and picking annually worsening A. *giganteana* invasions.

Numerous historical sources mention canebrakes growing in Native American old fields [45, 90, 99]. It seems likely that the vast and fertile canebrakes encountered by Euro-American settlers were, at least in part, the product of centuries of a Native American agroecology that served to use and maintain the productivity of fields and soils. Thus, Euro-American settlers were attracted to these long occupied bottomland “nodes”, in part, because of their improved productivity and ease of conversion over similar sites with no Native American occupation history.

### Native American legacies and persistence in landscape architecture

Based on our results, we hypothesize that the ecological legacies of Native Americans served as attractants and focal points of Euro-American settlement, and that these localized legacies helped to structure landscape-scale patterns of land use and boundaries that persist to the present. Using spatial overlay methods, we estimate that at least 15% of the contemporary boundary of the Sumter National Forest is composed of legacy property lines dating from the land grant surveys. Overlying the original property boundaries of tracts assembled into the National Forest starting in the 1930s, coincident boundaries between properties and land grants are even more prevalent. This is significant, because different land uses between pre-National Forest property owners often followed arbitrary property boundaries rather than natural landscape features [100]. Thus, a percentage of the contemporary distribution of land cover, e.g. between forest stand type and ages, can be attributed to original land grant boundaries. This effectively means that the hypothesized Euro-American settlement preferences for legacies of prehistoric Native American niche construction could have had a subtle but persistent effect on the structure of our contemporary landscape.

## Conclusions

We empirically demonstrate tangible historical links between millennial prehistoric Native American land use and the Euro-American settlement process for the Sumter National Forest in the South Carolina Piedmont. Although we cannot assert that Native American land use legacies directly caused the settlement location choices of Euro-Americans, the earliest Euro-Americans to settle the South Carolina Piedmont exhibited a preference for long term and late prehistoric Native American occupation sites above environmental characteristics including topographic position, slope, aspect, soil moisture, and solar radiation. Settlers were attracted to wide floodplains dominated by canebrakes (A. *giganteana*) that were most likely a legacy of Native American land use practices. We hypothesize that Euro-Americans were attracted to prehistoric Native American sites because they included land that was easier for travel, expedient to clear and plant, and because they were more productive. We attribute these qualities to localized ecological engineering activities of prehistoric Native Americans. Native Americans did not simply settle the most resource-rich environments, they altered them to suit agricultural land use; a process that took them multiple generations. Euro-Americans relying on maize and cattle took advantage of these previously engineered environments and were able to more quickly establish their agrarian economy.

Other examples highlighting the coevolution of landscape and society focus on places with demographic, if not cultural, continuity [14, 101]. Here, we suggest that the ecological legacies of Native Americans were significant and were generalized enough to confer an advantage to a demographically and culturally unrelated population of Euro-American settlers. If our interpretation is correct, this finding identifies a process whereby localized environmental modifications of prehistoric Native American populations indirectly contributed to large scale, contemporary landscape- and regional-level ecological inheritances. Significantly, it also dispels the notion of a wilderness frontier and suggests that the “humanized” portions of the landscape may have been crucial to settlement.

Over the last several decades, ecologists have begun to realize the importance of both historical contingency and past land use for understanding current and future ecological conditions [10, 102, 103]. At a minimum, our results point to continuity between millennia of Native American land use and the trajectory of the contemporary Piedmont landscapes. Because Native American land use may have created an underlying architecture that guided Euro-American settlement, prehistoric niche construction activities likely continue to influence household to regional level land use patterns as well as landscape-level resource management and conservation efforts such as Sumter National Forest. This research on the long term socioecological dynamics of the Calhoun CZO is a first effort to shed light on long term anthropogenic factors underlying the soil and hydrological degradation that followed closely on the heels of Euro-American settlement. Refining the spatial and temporal scope and resolution of land use history is important for explaining the spatiotemporal heterogeneity inherent in critical zone processes.

## Supporting information

S1 Table Data for the Cox proportional hazards model. Model covariates by sample unit Id.
(CSV)
